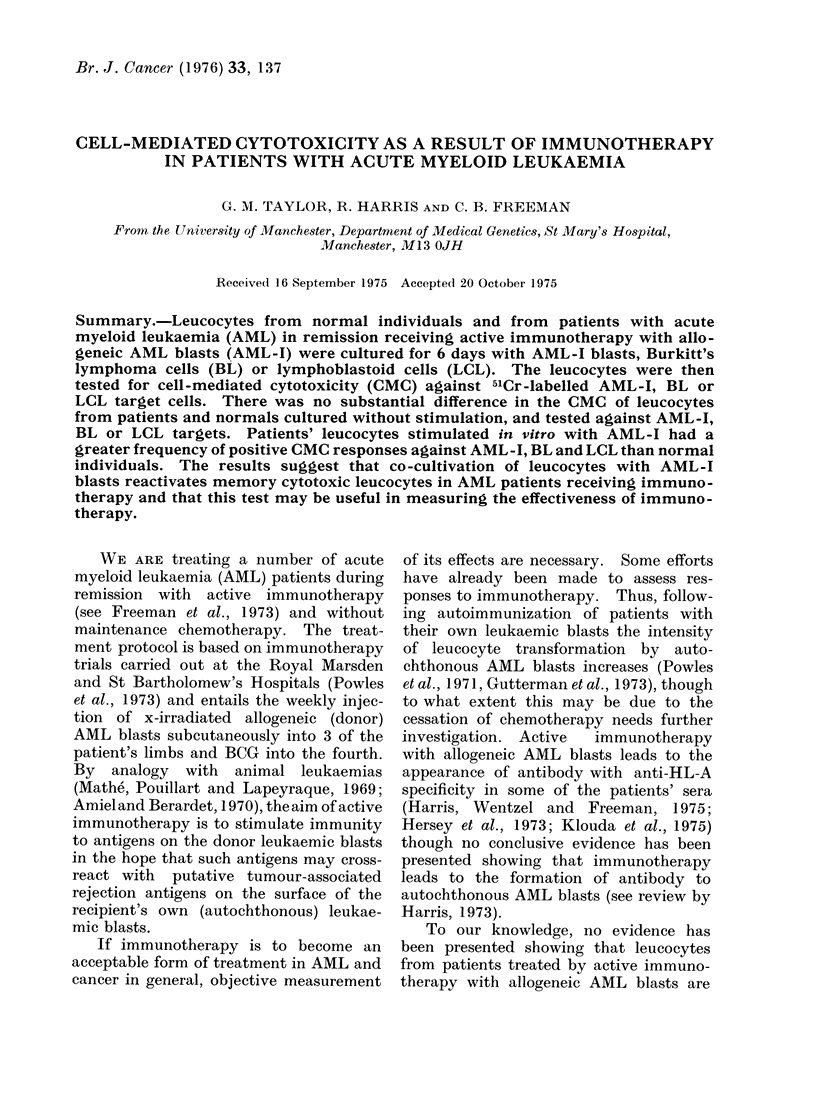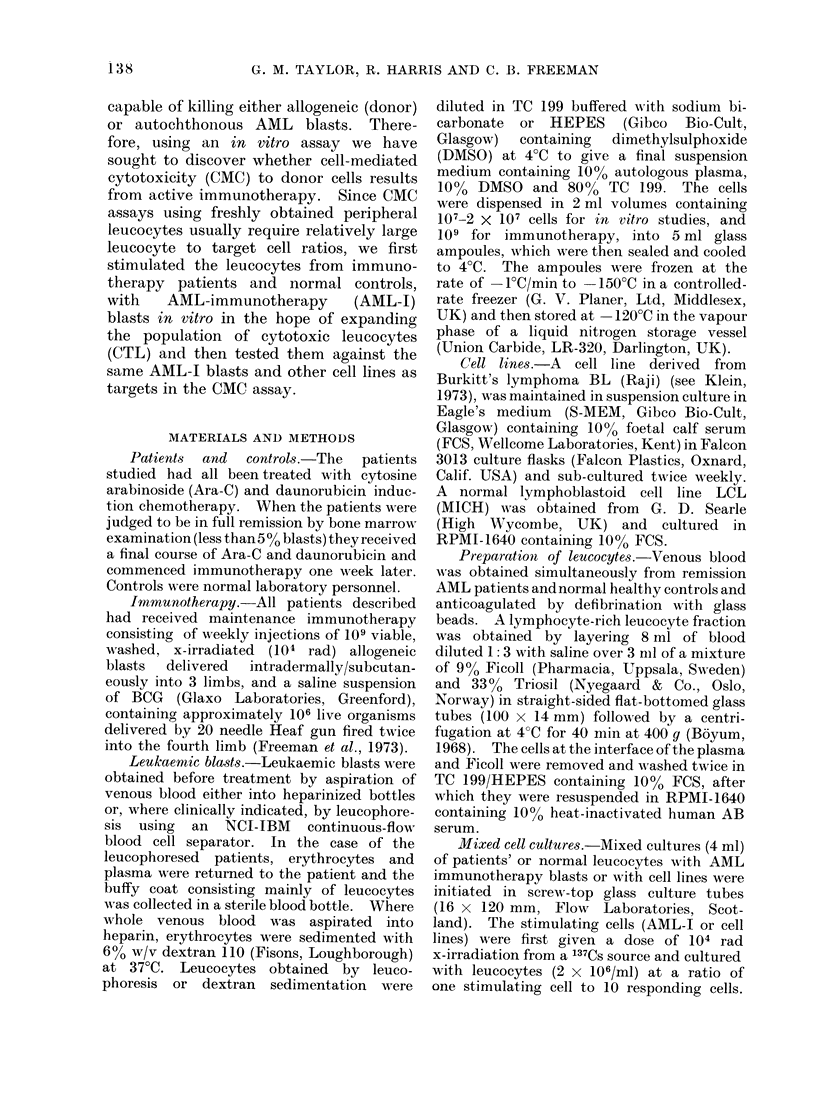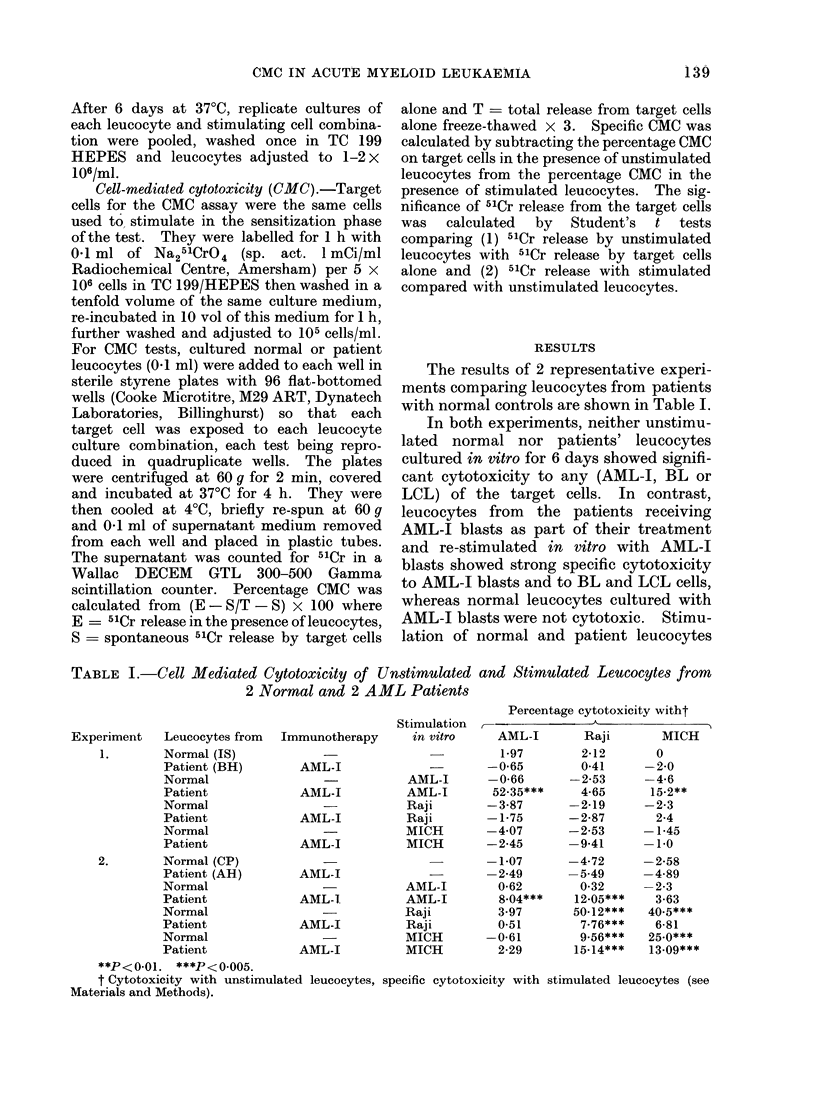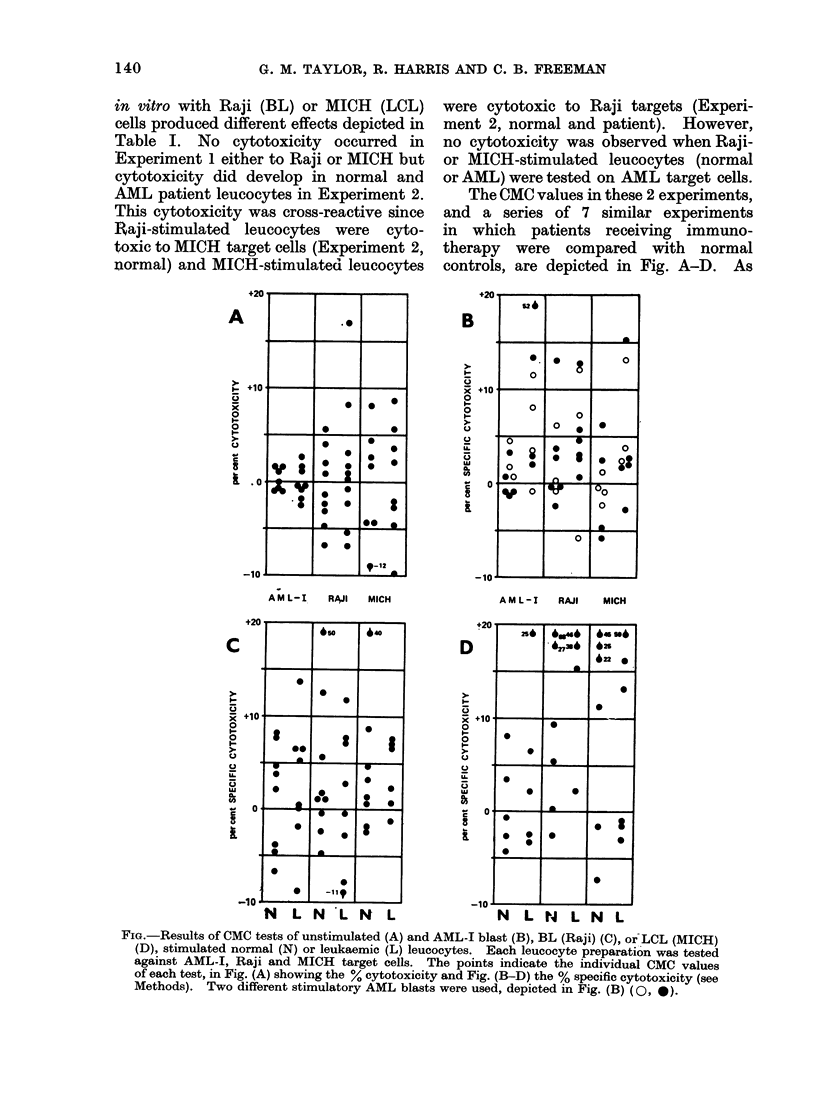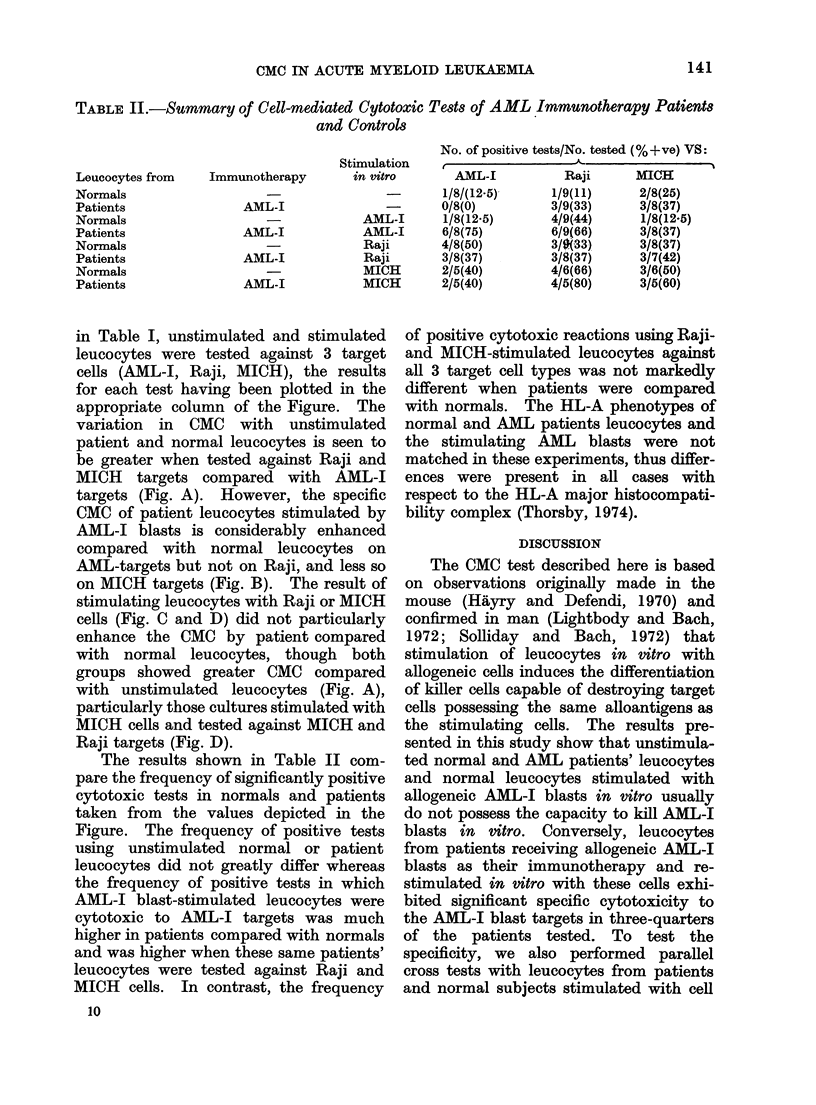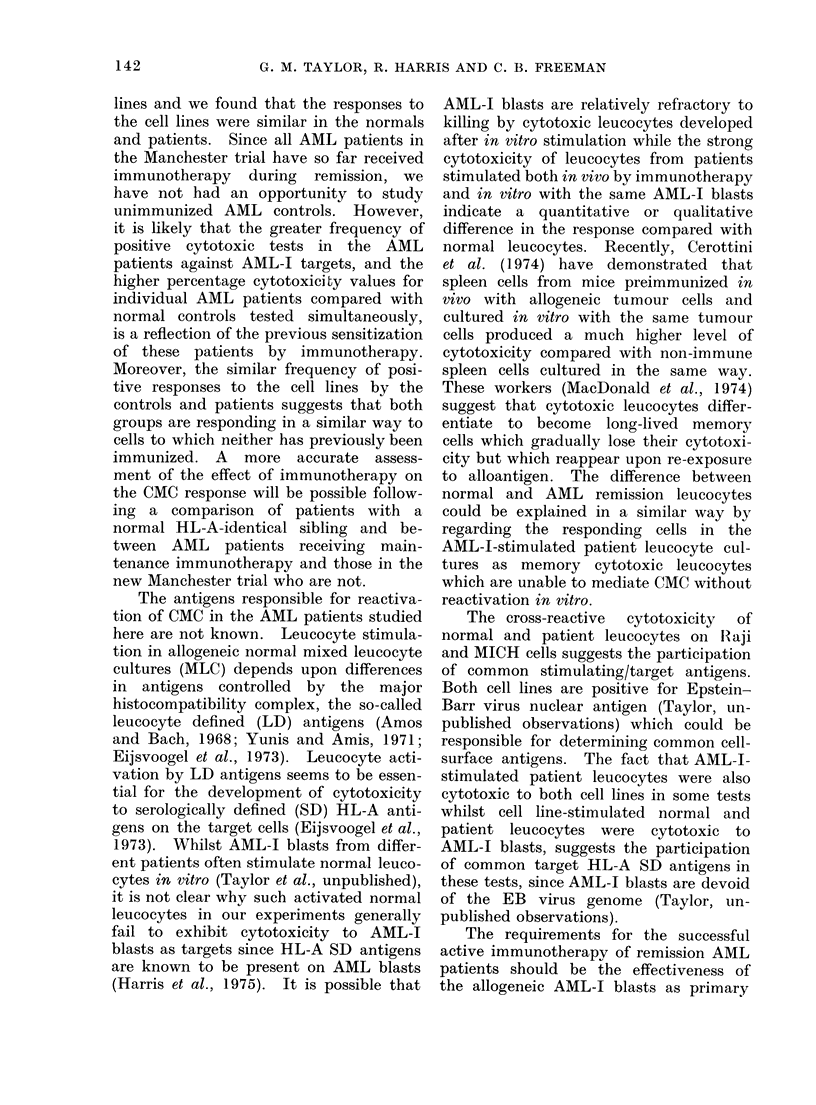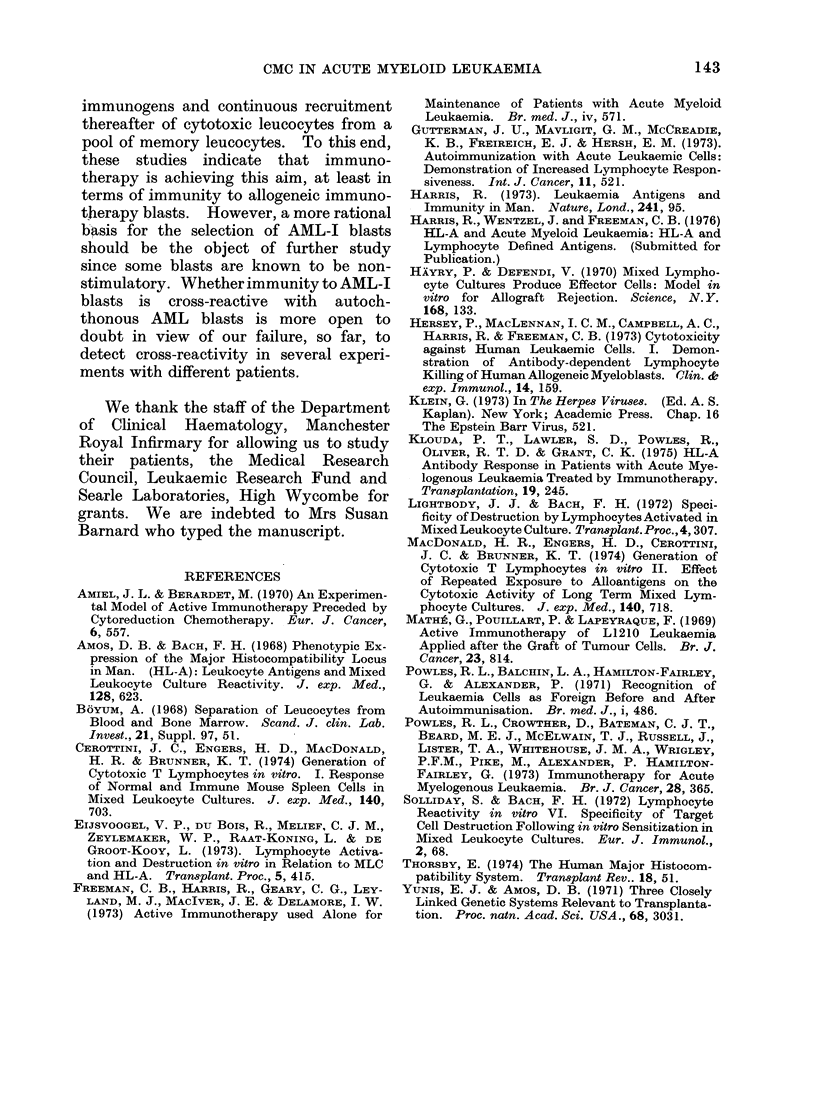# Cell-mediated cytotoxicity as a result of immunotherapy in patients with acute myeloid leukaemia.

**DOI:** 10.1038/bjc.1976.18

**Published:** 1976-02

**Authors:** G. M. Taylor, R. Harris, C. B. Freeman

## Abstract

Leucocytes from normal individuals and from patients with acute myeloid leukaemia (AML) in remission receiving active immunotherapy with allogeneic AML blasts (AML-I) were cultured for 6 days with AML-I blasts, Burkitt's lymphoma cells (BL) or lymphoblastoid cells (LCL). The leucocytes were then tested for cell-mediated cytotoxicity (CMC) against 51Cr-labelled AML-I, BL or LCL target cells. There was no substantial difference in the CMC of leucocytes from patients and normals cultured without stimulation, and tested against AML-I, BL or LCL targets. Patient's leucocytes stimulated in vitro with AML-I had a greater frequency of positive CMC responses against AML-I, BL and LCL than normal individuals. The results suggest that co-cultivation of leucocytes with AML-I blasts reactivates memory cytotoxic leucocytes in AML patients receiving immunotherapy and that this test may be useful in measuring the effectiveness of immunotherapy.


*Br. J. Cancer* (1976) **33**, 137

### CELL-MEDIATED CYTOTOXICITY AS A RESULT OF IMMUNOTHERAPY IN PATIENTS WITH ACUTE MYELOID LEUKAEMIA

G. M. TAYLOR, R. HARRIS AND C. B. FREEMAN

*From the University of Manchester, Department of Medical Genetics, St Mary's Hospital, Manchester, M13 0JH*

Received 16 September 1975 Accepted 20 October 1975

**Summary.—Leucocytes from normal individuals and from patients with acute myeloid leukaemia (AML) in remission receiving active immunotherapy with allogeneic AML blasts (AML-I) were cultured for 6 days with AML-I blasts, Burkitt's lymphoma cells (BL) or lymphoblastoid cells (LCL). The leucocytes were then tested for cell-mediated cytotoxicity (CMC) against $^{51}$Cr-labelled AML-I, BL or LCL target cells. There was no substantial difference in the CMC of leucocytes from patients and normals cultured without stimulation, and tested against AML-I, BL or LCL targets. Patients' leucocytes stimulated *in vitro* with AML-I had a greater frequency of positive CMC responses against AML-I, BL and LCL than normal individuals. The results suggest that co-cultivation of leucocytes with AML-I blasts reactivates memory cytotoxic leucocytes in AML patients receiving immunotherapy and that this test may be useful in measuring the effectiveness of immunotherapy.**

We are treating a number of acute myeloid leukaemia (AML) patients during remission with active immunotherapy (see Freeman *et al.*, 1973) and without maintenance chemotherapy. The treatment protocol is based on immunotherapy trials carried out at the Royal Marsden and St Bartholomew's Hospitals (Powles *et al.*, 1973) and entails the weekly injection of x-irradiated allogeneic (donor) AML blasts subcutaneously into 3 of the patient's limbs and BCG into the fourth. By analogy with animal leukaemias (Mathé, Pouillart and Lapeyraque, 1969; Amiel and Berardet, 1970), the aim of active immunotherapy is to stimulate immunity to antigens on the donor leukaemic blasts in the hope that such antigens may cross-react with putative tumour-associated rejection antigens on the surface of the recipient's own (autochthonous) leukaemic blasts.

If immunotherapy is to become an acceptable form of treatment in AML and cancer in general, objective measurement of its effects are necessary. Some efforts have already been made to assess responses to immunotherapy. Thus, following autoimmunization of patients with their own leukaemic blasts the intensity of leucocyte transformation by autochthonous AML blasts increases (Powles *et al.*, 1971, Gutterman *et al.*, 1973), though to what extent this may be due to the cessation of chemotherapy needs further investigation. Active immunotherapy with allogeneic AML blasts leads to the appearance of antibody with anti-HL-A specificity in some of the patients' sera (Harris, Wentzel and Freeman, 1975; Hersey *et al.*, 1973; Klouda *et al.*, 1975) though no conclusive evidence has been presented showing that immunotherapy leads to the formation of antibody to autochthonous AML blasts (see review by Harris, 1973).

To our knowledge, no evidence has been presented showing that leucocytes from patients treated by active immunotherapy with allogeneic AML blasts are capable of killing either allogeneic (donor) or autochthonous AML blasts. Therefore, using an *in vitro* assay we have sought to discover whether cell-mediated cytotoxicity (CMC) to donor cells results from active immunotherapy. Since CMC assays using freshly obtained peripheral leucocytes usually require relatively large leucocyte to target cell ratios, we first stimulated the leucocytes from immunotherapy patients and normal controls, with AML-immunotherapy (AML-I) blasts *in vitro* in the hope of expanding the population of cytotoxic leucocytes (CTL) and then tested them against the same AML-I blasts and other cell lines as targets in the CMC assay.

### MATERIALS AND METHODS

*Patients and controls.*—The patients studied had all been treated with cytosine arabinoside (Ara-C) and daunorubicin induction chemotherapy. When the patients were judged to be in full remission by bone marrow examination (less than 5% blasts) they received a final course of Ara-C and daunorubicin and commenced immunotherapy one week later. Controls were normal laboratory personnel.

*Immunotherapy.*—All patients described had received maintenance immunotherapy consisting of weekly injections of $10^9$ viable, washed, x-irradiated ($10^4$ rad) allogeneic blasts delivered intradermally/subcutaneously into 3 limbs, and a saline suspension of BCG (Glaxo Laboratories, Greenford), containing approximately $10^6$ live organisms delivered by 20 needle Heaf gun fired twice into the fourth limb (Freeman *et al.*, 1973).

*Leukaemic blasts.*—Leukaemic blasts were obtained before treatment by aspiration of venous blood either into heparinized bottles or, where clinically indicated, by leucophoresis using an NCI-IBM continuous-flow blood cell separator. In the case of the leucophoresed patients, erythrocytes and plasma were returned to the patient and the buffy coat consisting mainly of leucocytes was collected in a sterile blood bottle. Where whole venous blood was aspirated into heparin, erythrocytes were sedimented with 6% w/v dextran 110 (Fisons, Loughborough) at 37°C. Leucocytes obtained by leucophoresis or dextran sedimentation were diluted in TC 199 buffered with sodium bicarbonate or HEPES (Gibco Bio-Cult, Glasgow) containing dimethylsulphoxide (DMSO) at 4°C to give a final suspension medium containing 10% autologous plasma, 10% DMSO and 80% TC 199. The cells were dispensed in 2 ml volumes containing $10^7$–$2 \times 10^7$ cells for *in vitro* studies, and $10^9$ for immunotherapy, into 5 ml glass ampoules, which were then sealed and cooled to 4°C. The ampoules were frozen at the rate of −1°C/min to −150°C in a controlled-rate freezer (G. V. Planer, Ltd, Middlesex, UK) and then stored at −120°C in the vapour phase of a liquid nitrogen storage vessel (Union Carbide, LR-320, Darlington, UK).

*Cell lines.*—A cell line derived from Burkitt's lymphoma BL (Raji) (see Klein, 1973), was maintained in suspension culture in Eagle's medium (S-MEM, Gibco Bio-Cult, Glasgow) containing 10% foetal calf serum (FCS, Wellcome Laboratories, Kent) in Falcon 3013 culture flasks (Falcon Plastics, Oxnard, Calif. USA) and sub-cultured twice weekly. A normal lymphoblastoid cell line LCL (MICH) was obtained from G. D. Searle (High Wycombe, UK) and cultured in RPMI-1640 containing 10% FCS.

*Preparation of leucocytes.*—Venous blood was obtained simultaneously from remission AML patients and normal healthy controls and anticoagulated by defibrination with glass beads. A lymphocyte-rich leucocyte fraction was obtained by layering 8 ml of blood diluted 1 : 3 with saline over 3 ml of a mixture of 9% Ficoll (Pharmacia, Uppsala, Sweden) and 33% Triosil (Nyegaard & Co., Oslo, Norway) in straight-sided flat-bottomed glass tubes (100 × 14 mm) followed by a centrifugation at 4°C for 40 min at 400 *g* (Böyum, 1968). The cells at the interface of the plasma and Ficoll were removed and washed twice in TC 199/HEPES containing 10% FCS, after which they were resuspended in RPMI-1640 containing 10% heat-inactivated human AB serum.

*Mixed cell cultures.*—Mixed cultures (4 ml) of patients' or normal leucocytes with AML immunotherapy blasts or with cell lines were initiated in screw-top glass culture tubes (16 × 120 mm, Flow Laboratories, Scotland). The stimulating cells (AML-I or cell lines) were first given a dose of $10^4$ rad x-irradiation from a $^{137}Cs$ source and cultured with leucocytes ($2 \times 10^6$/ml) at a ratio of one stimulating cell to 10 responding cells.

After 6 days at 37°C, replicate cultures of each leucocyte and stimulating cell combination were pooled, washed once in TC 199 HEPES and leucocytes adjusted to 1–2 × $10^6$/ml.

*Cell-mediated cytotoxicity* (*CMC*).—Target cells for the CMC assay were the same cells used to stimulate in the sensitization phase of the test. They were labelled for 1 h with 0·1 ml of $Na_2{}^{51}CrO_4$ (sp. act. 1 mCi/ml Radiochemical Centre, Amersham) per 5 × $10^6$ cells in TC 199/HEPES then washed in a tenfold volume of the same culture medium, re-incubated in 10 vol of this medium for 1 h, further washed and adjusted to $10^5$ cells/ml. For CMC tests, cultured normal or patient leucocytes (0·1 ml) were added to each well in sterile styrene plates with 96 flat-bottomed wells (Cooke Microtitre, M29 ART, Dynatech Laboratories, Billinghurst) so that each target cell was exposed to each leucocyte culture combination, each test being reproduced in quadruplicate wells. The plates were centrifuged at 60 *g* for 2 min, covered and incubated at 37°C for 4 h. They were then cooled at 4°C, briefly re-spun at 60 *g* and 0·1 ml of supernatant medium removed from each well and placed in plastic tubes. The supernatant was counted for $^{51}Cr$ in a Wallac DECEM GTL 300–500 Gamma scintillation counter. Percentage CMC was calculated from (E − S/T − S) × 100 where E = $^{51}Cr$ release in the presence of leucocytes, S = spontaneous $^{51}Cr$ release by target cells alone and T = total release from target cells alone freeze-thawed × 3. Specific CMC was calculated by subtracting the percentage CMC on target cells in the presence of unstimulated leucocytes from the percentage CMC in the presence of stimulated leucocytes. The significance of $^{51}Cr$ release from the target cells was calculated by Student's *t* tests comparing (1) $^{51}Cr$ release by unstimulated leucocytes with $^{51}Cr$ release by target cells alone and (2) $^{51}Cr$ release with stimulated compared with unstimulated leucocytes.

### RESULTS

The results of 2 representative experiments comparing leucocytes from patients with normal controls are shown in Table I.

In both experiments, neither unstimulated normal nor patients' leucocytes cultured *in vitro* for 6 days showed significant cytotoxicity to any (AML-I, BL or LCL) of the target cells. In contrast, leucocytes from the patients receiving AML-I blasts as part of their treatment and re-stimulated *in vitro* with AML-I blasts showed strong specific cytotoxicity to AML-I blasts and to BL and LCL cells, whereas normal leucocytes cultured with AML-I blasts were not cytotoxic. Stimulation of normal and patient leucocytes

TABLE I.—*Cell Mediated Cytotoxicity of Unstimulated and Stimulated Leucocytes from 2 Normal and 2 AML Patients*

| Experiment | Leucocytes from | Immunotherapy | Stimulation *in vitro* | Percentage cytotoxicity with† | | |
|---|---|---|---|---|---|---|
| | | | | AML-I | Raji | MICH |
| 1. | Normal (IS) | — | — | 1·97 | 2·12 | 0 |
| | Patient (BH) | AML-I | — | −0·65 | 0·41 | −2·0 |
| | Normal | — | AML-I | −0·66 | −2·53 | −4·6 |
| | Patient | AML-I | AML-I | 52·35*** | 4·65 | 15·2** |
| | Normal | — | Raji | −3·87 | −2·19 | −2·3 |
| | Patient | AML-I | Raji | −1·75 | −2·87 | 2·4 |
| | Normal | — | MICH | −4·07 | −2·53 | −1·45 |
| | Patient | AML-I | MICH | −2·45 | −9·41 | −1·0 |
| 2. | Normal (CP) | — | — | −1·07 | −4·72 | −2·58 |
| | Patient (AH) | AML-I | — | −2·49 | −5·49 | −4·89 |
| | Normal | — | AML-I | 0·62 | 0·32 | −2·3 |
| | Patient | AML-I | AML-I | 8·04*** | 12·05*** | 3·63 |
| | Normal | — | Raji | 3·97 | 50·12*** | 40·5*** |
| | Patient | AML-I | Raji | 0·51 | 7·76*** | 6·81 |
| | Normal | — | MICH | −0·61 | 9·56*** | 25·0*** |
| | Patient | AML-I | MICH | 2·29 | 15·14*** | 13·09*** |

$^{**}P<0·01$. $^{***}P<0·005$.

† Cytotoxicity with unstimulated leucocytes, specific cytotoxicity with stimulated leucocytes (see Materials and Methods).

*in vitro* with Raji (BL) or MICH (LCL) cells produced different effects depicted in Table I. No cytotoxicity occurred in Experiment 1 either to Raji or MICH but cytotoxicity did develop in normal and AML patient leucocytes in Experiment 2. This cytotoxicity was cross-reactive since Raji-stimulated leucocytes were cytotoxic to MICH target cells (Experiment 2, normal) and MICH-stimulated leucocytes were cytotoxic to Raji targets (Experiment 2, normal and patient). However, no cytotoxicity was observed when Raji- or MICH-stimulated leucocytes (normal or AML) were tested on AML target cells.

The CMC values in these 2 experiments, and a series of 7 similar experiments in which patients receiving immunotherapy were compared with normal controls, are depicted in Fig. A–D. As

Fig.—Results of CMC tests of unstimulated (A) and AML-I blast (B), BL (Raji) (C), or LCL (MICH) (D), stimulated normal (N) or leukaemic (L) leucocytes. Each leucocyte preparation was tested against AML-I, Raji and MICH target cells. The points indicate the individual CMC values of each test, in Fig. (A) showing the % cytotoxicity and Fig. (B–D) the % specific cytotoxicity (see Methods). Two different stimulatory AML blasts were used, depicted in Fig. (B) (○, ●).

TABLE II.—*Summary of Cell-mediated Cytotoxic Tests of AML Immunotherapy Patients and Controls*

| Leucocytes from | Immunotherapy | Stimulation *in vitro* | No. of positive tests/No. tested (%+ve) VS: AML-I | Raji | MICH |
|---|---|---|---|---|---|
| Normals | — | — | 1/8/(12·5) | 1/9(11) | 2/8(25) |
| Patients | AML-I | — | 0/8(0) | 3/9(33) | 3/8(37) |
| Normals | — | AML-I | 1/8(12·5) | 4/9(44) | 1/8(12·5) |
| Patients | AML-I | AML-I | 6/8(75) | 6/9(66) | 3/8(37) |
| Normals | — | Raji | 4/8(50) | 3/9(33) | 3/8(37) |
| Patients | AML-I | Raji | 3/8(37) | 3/8(37) | 3/7(42) |
| Normals | — | MICH | 2/5(40) | 4/6(66) | 3/6(50) |
| Patients | AML-I | MICH | 2/5(40) | 4/5(80) | 3/5(60) |

in Table I, unstimulated and stimulated leucocytes were tested against 3 target cells (AML-I, Raji, MICH), the results for each test having been plotted in the appropriate column of the Figure. The variation in CMC with unstimulated patient and normal leucocytes is seen to be greater when tested against Raji and MICH targets compared with AML-I targets (Fig. A). However, the specific CMC of patient leucocytes stimulated by AML-I blasts is considerably enhanced compared with normal leucocytes on AML-targets but not on Raji, and less so on MICH targets (Fig. B). The result of stimulating leucocytes with Raji or MICH cells (Fig. C and D) did not particularly enhance the CMC by patient compared with normal leucocytes, though both groups showed greater CMC compared with unstimulated leucocytes (Fig. A), particularly those cultures stimulated with MICH cells and tested against MICH and Raji targets (Fig. D).

The results shown in Table II compare the frequency of significantly positive cytotoxic tests in normals and patients taken from the values depicted in the Figure. The frequency of positive tests using unstimulated normal or patient leucocytes did not greatly differ whereas the frequency of positive tests in which AML-I blast-stimulated leucocytes were cytotoxic to AML-I targets was much higher in patients compared with normals and was higher when these same patients' leucocytes were tested against Raji and MICH cells. In contrast, the frequency of positive cytotoxic reactions using Raji- and MICH-stimulated leucocytes against all 3 target cell types was not markedly different when patients were compared with normals. The HL-A phenotypes of normal and AML patients leucocytes and the stimulating AML blasts were not matched in these experiments, thus differences were present in all cases with respect to the HL-A major histocompatibility complex (Thorsby, 1974).

### DISCUSSION

The CMC test described here is based on observations originally made in the mouse (Häyry and Defendi, 1970) and confirmed in man (Lightbody and Bach, 1972; Solliday and Bach, 1972) that stimulation of leucocytes *in vitro* with allogeneic cells induces the differentiation of killer cells capable of destroying target cells possessing the same alloantigens as the stimulating cells. The results presented in this study show that unstimulated normal and AML patients' leucocytes and normal leucocytes stimulated with allogeneic AML-I blasts *in vitro* usually do not possess the capacity to kill AML-I blasts *in vitro*. Conversely, leucocytes from patients receiving allogeneic AML-I blasts as their immunotherapy and restimulated *in vitro* with these cells exhibited significant specific cytotoxicity to the AML-I blast targets in three-quarters of the patients tested. To test the specificity, we also performed parallel cross tests with leucocytes from patients and normal subjects stimulated with cell lines and we found that the responses to the cell lines were similar in the normals and patients. Since all AML patients in the Manchester trial have so far received immunotherapy during remission, we have not had an opportunity to study unimmunized AML controls. However, it is likely that the greater frequency of positive cytotoxic tests in the AML patients against AML-I targets, and the higher percentage cytotoxicity values for individual AML patients compared with normal controls tested simultaneously, is a reflection of the previous sensitization of these patients by immunotherapy. Moreover, the similar frequency of positive responses to the cell lines by the controls and patients suggests that both groups are responding in a similar way to cells to which neither has previously been immunized. A more accurate assessment of the effect of immunotherapy on the CMC response will be possible following a comparison of patients with a normal HL-A-identical sibling and between AML patients receiving maintenance immunotherapy and those in the new Manchester trial who are not.

The antigens responsible for reactivation of CMC in the AML patients studied here are not known. Leucocyte stimulation in allogeneic normal mixed leucocyte cultures (MLC) depends upon differences in antigens controlled by the major histocompatibility complex, the so-called leucocyte defined (LD) antigens (Amos and Bach, 1968; Yunis and Amis, 1971; Eijsvoogel *et al.*, 1973). Leucocyte activation by LD antigens seems to be essential for the development of cytotoxicity to serologically defined (SD) HL-A antigens on the target cells (Eijsvoogel *et al.*, 1973). Whilst AML-I blasts from different patients often stimulate normal leucocytes *in vitro* (Taylor *et al.*, unpublished), it is not clear why such activated normal leucocytes in our experiments generally fail to exhibit cytotoxicity to AML-I blasts as targets since HL-A SD antigens are known to be present on AML blasts (Harris *et al.*, 1975). It is possible that AML-I blasts are relatively refractory to killing by cytotoxic leucocytes developed after *in vitro* stimulation while the strong cytotoxicity of leucocytes from patients stimulated both *in vivo* by immunotherapy and *in vitro* with the same AML-I blasts indicate a quantitative or qualitative difference in the response compared with normal leucocytes. Recently, Cerottini *et al.* (1974) have demonstrated that spleen cells from mice preimmunized *in vivo* with allogeneic tumour cells and cultured *in vitro* with the same tumour cells produced a much higher level of cytotoxicity compared with non-immune spleen cells cultured in the same way. These workers (MacDonald *et al.*, 1974) suggest that cytotoxic leucocytes differentiate to become long-lived memory cells which gradually lose their cytotoxicity but which reappear upon re-exposure to alloantigen. The difference between normal and AML remission leucocytes could be explained in a similar way by regarding the responding cells in the AML-I-stimulated patient leucocyte cultures as memory cytotoxic leucocytes which are unable to mediate CMC without reactivation *in vitro*.

The cross-reactive cytotoxicity of normal and patient leucocytes on Raji and MICH cells suggests the participation of common stimulating/target antigens. Both cell lines are positive for Epstein–Barr virus nuclear antigen (Taylor, unpublished observations) which could be responsible for determining common cell-surface antigens. The fact that AML-I-stimulated patient leucocytes were also cytotoxic to both cell lines in some tests whilst cell line-stimulated normal and patient leucocytes were cytotoxic to AML-I blasts, suggests the participation of common target HL-A SD antigens in these tests, since AML-I blasts are devoid of the EB virus genome (Taylor, unpublished observations).

The requirements for the successful active immunotherapy of remission AML patients should be the effectiveness of the allogeneic AML-I blasts as primary immunogens and continuous recruitment thereafter of cytotoxic leucocytes from a pool of memory leucocytes. To this end, these studies indicate that immunotherapy is achieving this aim, at least in terms of immunity to allogeneic immunotherapy blasts. However, a more rational basis for the selection of AML-I blasts should be the object of further study since some blasts are known to be non-stimulatory. Whether immunity to AML-I blasts is cross-reactive with autochthonous AML blasts is more open to doubt in view of our failure, so far, to detect cross-reactivity in several experiments with different patients.

We thank the staff of the Department of Clinical Haematology, Manchester Royal Infirmary for allowing us to study their patients, the Medical Research Council, Leukaemic Research Fund and Searle Laboratories, High Wycombe for grants. We are indebted to Mrs Susan Barnard who typed the manuscript.

REFERENCES

AMIEL, J. L. & BERARDET, M. (1970) An Experimental Model of Active Immunotherapy Preceded by Cytoreduction Chemotherapy. *Eur. J. Cancer*, **6**, 557.

AMOS, D. B. & BACH, F. H. (1968) Phenotypic Expression of the Major Histocompatibility Locus in Man. (HL-A): Leukocyte Antigens and Mixed Leukocyte Culture Reactivity. *J. exp. Med.*, **128**, 623.

BÖYUM, A. (1968) Separation of Leucocytes from Blood and Bone Marrow. *Scand. J. clin. Lab. Invest.*, **21**, Suppl. 97, 51.

CEROTTINI, J. C., ENGERS, H. D., MACDONALD, H. R. & BRUNNER, K. T. (1974) Generation of Cytotoxic T Lymphocytes *in vitro*. I. Response of Normal and Immune Mouse Spleen Cells in Mixed Leukocyte Cultures. *J. exp. Med.*, **140**, 703.

EIJSVOOGEL, V. P., DU BOIS, R., MELIEF, C. J. M., ZEYLEMAKER, W. P., RAAT-KONING, L. & DE GROOT-KOOY, L. (1973). Lymphocyte Activation and Destruction *in vitro* in Relation to MLC and HL-A. *Transplant. Proc.*, **5**, 415.

FREEMAN, C. B., HARRIS, R., GEARY, C. G., LEYLAND, M. J., MACIVER, J. E. & DELAMORE, I. W. (1973) Active Immunotherapy used Alone for Maintenance of Patients with Acute Myeloid Leukaemia. *Br. med. J.*, iv, 571.

GUTTERMAN, J. U., MAVLIGIT, G. M., MCCREADIE, K. B., FREIREICH, E. J. & HERSH, E. M. (1973). Autoimmunization with Acute Leukaemic Cells: Demonstration of Increased Lymphocyte Responsiveness. *Int. J. Cancer*, **11**, 521.

HARRIS, R. (1973). Leukaemia Antigens and Immunity in Man. *Nature, Lond.*, **241**, 95.

HARRIS, R., WENTZEL, J. and FREEMAN, C. B. (1976) HL-A and Acute Myeloid Leukaemia: HL-A and Lymphocyte Defined Antigens. (Submitted for Publication.)

HÄYRY, P. & DEFENDI, V. (1970) Mixed Lymphocyte Cultures Produce Effector Cells: Model *in vitro* for Allograft Rejection. *Science, N.Y.* **168**, 133.

HERSEY, P., MACLENNAN, I. C. M., CAMPBELL, A. C., HARRIS, R. & FREEMAN, C. B. (1973) Cytotoxicity against Human Leukaemic Cells. I. Demonstration of Antibody-dependent Lymphocyte Killing of Human Allogeneic Myeloblasts. *Clin. & exp. Immunol.*, **14**, 159.

KLEIN, G. (1973) In *The Herpes Viruses*. (Ed. A. S. Kaplan). New York; Academic Press. Chap. 16 The Epstein Barr Virus, 521.

KLOUDA, P. T., LAWLER, S. D., POWLES, R., OLIVER, R. T. D. & GRANT, C. K. (1975) HL-A Antibody Response in Patients with Acute Myelogenous Leukaemia Treated by Immunotherapy. *Transplantation*, **19**, 245.

LIGHTBODY, J. J. & BACH, F. H. (1972) Specificity of Destruction by Lymphocytes Activated in Mixed Leukocyte Culture. *Transplant. Proc.*, **4**, 307.

MACDONALD, H. R., ENGERS, H. D., CEROTTINI, J. C. & BRUNNER, K. T. (1974) Generation of Cytotoxic T Lymphocytes *in vitro* II. Effect of Repeated Exposure to Alloantigens on the Cytotoxic Activity of Long Term Mixed Lymphocyte Cultures. *J. exp. Med.*, **140**, 718.

MATHÉ, G., POUILLART, P. & LAPEYRAQUE, F. (1969) Active Immunotherapy of L1210 Leukaemia Applied after the Graft of Tumour Cells. *Br. J. Cancer*, **23**, 814.

POWLES, R. L., BALCHIN, L. A., HAMILTON-FAIRLEY, G. & ALEXANDER, P. (1971) Recognition of Leukaemia Cells as Foreign Before and After Autoimmunisation. *Br. med. J.*, i, 486.

POWLES, R. L., CROWTHER, D., BATEMAN, C. J. T., BEARD, M. E. J., MCELWAIN, T. J., RUSSELL, J., LISTER, T. A., WHITEHOUSE, J. M. A., WRIGLEY, P.F.M., PIKE, M., ALEXANDER, P. HAMILTON-FAIRLEY, G. (1973) Immunotherapy for Acute Myelogenous Leukaemia. *Br. J. Cancer*, **28**, 365.

SOLLIDAY, S. & BACH, F. H. (1972) Lymphocyte Reactivity *in vitro* VI. Specificity of Target Cell Destruction Following *in vitro* Sensitization in Mixed Leukocyte Cultures. *Eur. J. Immunol.*, **2**, 68.

THORSBY, E. (1974) The Human Major Histocompatibility System. *Transplant Rev.*. **18**, 51.

YUNIS, E. J. & AMOS, D. B. (1971) Three Closely Linked Genetic Systems Relevant to Transplantation. *Proc. natn. Acad. Sci. USA.*, **68**, 3031.